# A Second Life For Scraps: Making Biogas From Food Waste

**DOI:** 10.1289/ehp.123-A180

**Published:** 2015-07-01

**Authors:** Richard Dahl

**Affiliations:** Richard Dahl, a freelance writer in Boston, MA, also writes periodically for the Massachusetts Institute of Technology.

In 2010 an estimated 31% of the food in U.S. stores and homes went uneaten, and Americans shipped approximately 34 million tons of food waste to landfills.^1^ When food decomposes under anaerobic conditions—for instance, buried beneath other waste in a landfill—it produces methane, a highly potent greenhouse gas.^1^ According to the U.S. Environmental Protection Agency, landfills are the third largest producer of methane in the United States, accounting for about 18% of methane emissions in 2013.^2^

In recent years U.S. policy makers have begun to take action to reduce the flow of food into landfills, in part to stem greenhouse gas emissions but also to save businesses the cost of disposal. In 2011 Connecticut became the first state to initiate such action, prohibiting the landfilling of food waste by entities that generate at least two tons per week and are located within 20 miles of a food-recycling facility.^3^ In 2012 Vermont initiated a ban similar to Connecticut’s but included a phase-in schedule in which smaller waste generators will face bans in coming years.^3^ In October 2014 Massachusetts launched the toughest, most ambitious such plan in the country by banning the landfilling of food waste by all entities that produce at least one ton of food waste per week.^4^ And as of 1 January 2015, Seattle requires that food waste make up no more than 10% by volume of any resident’s or business’s trash.^5^

**Figure:**
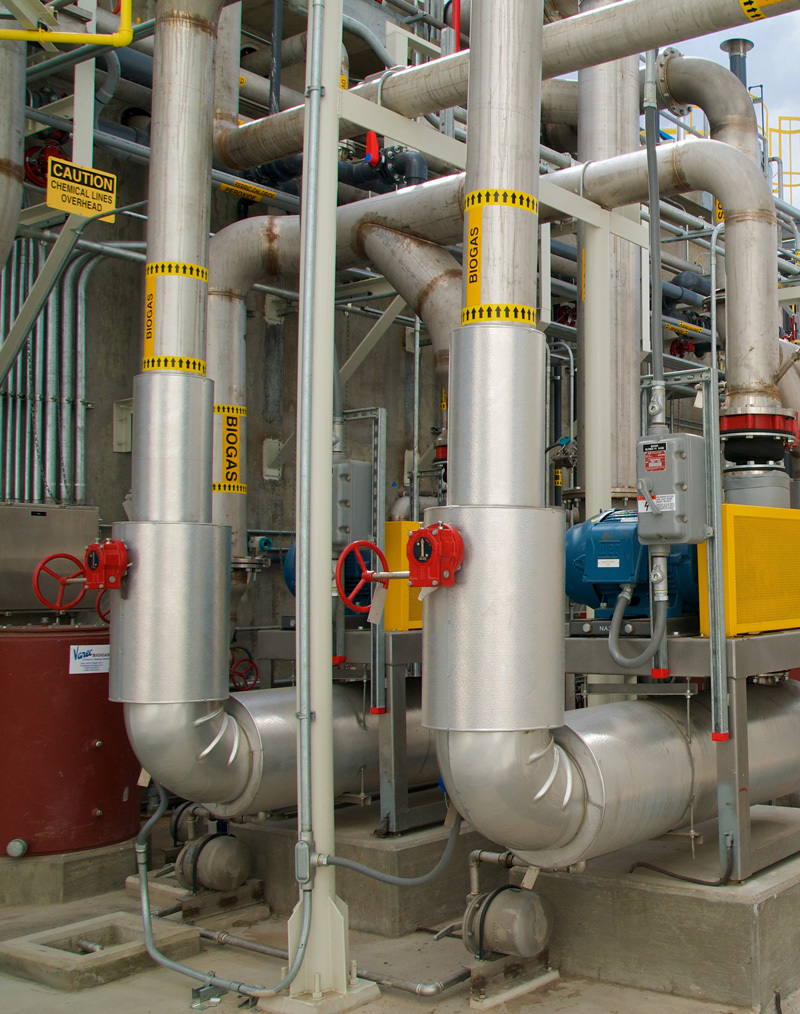
Biodigesters use organic waste to produce methane, a relatively clean fuel. Since 2013 Kroger grocery stores have sent this facility all the food they can’t sell or donate. © FEED Resource Recovery, Inc

But if food is banned from landfills, where can it go? The primary options have been to give unsold food to food pantries or ship it to composting facilities. But recently anaerobic digestion has been drawing attention as a method that can not only accomplish greenhouse gas reduction but also use the food waste as an energy source.

## New Attention to an Old Technology

Anaerobic digestion is not a new technology. According to the American Biogas Council, 1,241 wastewater treatment plants in the United States have anaerobic digesters, as do 239 farms.^6^ What’s new, at least in this country, is the inclusion of food waste alongside wastewater and livestock manure—a move prompted by the new governmental restrictions on landfilling. Elsewhere in the world, the inclusion of food in anaerobic digesters is well established.

The process of anaerobic digestion is akin to the digestive processes that occur within mammals. Microorganisms break down biodegradable material in the absence of oxygen (composting, by comparison, requires aerating the pile to keep the process aerobic^7^). One of the end products is biogas, composed mostly of methane and carbon dioxide, with lesser amounts of water and gases such as nitrogen and hydrogen sulfide.^6^ Another end product is a residual digestate, a solid, nutrient-rich substance that can be used as fertilizer.^6^

Digesters take various forms, but they are essentially tanks with the capability of capturing methane that is produced in the process and piping the gas to a generator that converts it into electrical power and heat.^6^ Biogas can be burned onsite or processed into natural gas or liquid transportation fuels. Refined biogas is considered a clean fuel, says Anthony Fiore, energy program director at the New York City Department of Environmental Protection (DEP). “As long as the methane is used beneficially, it is ‘clean,’” Fiore explains. “However, if the methane is simply flared to the atmosphere, then it is considered a greenhouse gas.”

In Massachusetts, the first anaerobic digesters to utilize food waste appeared three years ago when two dairy farms installed them and began accepting food waste from area producers. One of them, Barstow’s Longview Farm, produces milk for Cabot Creamery in West Springfield. The farm feeds its digester with a mixture of cow manure and buttermilk by-product from the creamery. The resulting methane creates enough electricity to power the farm, and the excess is sold to the creamery.^8^

In 2014 the Massachusetts Water Resource Authority launched a pilot project to include food waste in the existing anaerobic digester at the Deer Island Sewage Treatment Plant, and that same month a food-waste processing operation went into business at a landfill near New Bedford.^9^ That operation, the CRMC Dartmouth Bioenergy Facility, is linked to an existing gas-to-energy facility that generates power from landfill methane. Anton Finelli, a principal in CommonWealth Resource Management Corporation, which owns the operation, says the company saw opportunity in the landfill ban.

“We realized it would be to our benefit to have the ability right at the landfill to process source-separated organics [i.e., organic matter that consumers have sorted at curbside] that the landfill itself would sooner or later no longer be able to take,” Finelli says. “Our principle motivation here was to have another way to generate a biogas for use at our landfill gas-to-energy facility. And that part of our pilot project has been very successful. The rate of biogas production is greater than we projected per volume of input.”

Finelli says the company is planning to expand the operation from its current capacity of 100,000 gallons to 1 million gallons. “The expansion will require another round of permitting, a process which could take a year by itself,” Finelli says. “Construction would add another six months if well-timed. So, the scaled-up facility is at least another eighteen months away.”

## Feasibility Testing in New York

New York City is also taking a serious look at anaerobic digestion as part of its ambitious plan of requiring commercial food establishments over a certain size to recycle their organic waste starting 1 July 2015.^10^ The New York City DEP just completed a one-year pilot project adding food waste to an existing digester at the Newtown Creek Wastewater Treatment Plant in Brooklyn.

The food waste had been collected at city schools and urban farmers’ markets, then trucked to a processing facility where it was liquefied into “bioslurry.” Fiore points out that the scale of the pilot project was tiny—the daily addition of bioslurry amounted to less than 500 gallons, while each of the plant’s eight digesters holds 3 million gallons—so measurement of its overall impact on methane production was impossible. The purpose of the pilot project, he says, was to test logistics, and to that extent, it was a success.

The city will initiate a significantly larger three-year demonstration project at Newtown Creek starting at the end of 2015. This project is initially set to take in 50 tons of bioslurry per day, ramping up to 250 tons by the end of the project.

“At that scale, it’s pretty much unprecedented in this country,” Fiore says. “If it’s successful, we think we have enough capacity in this plant to take up to 500 tons per day. And if we were able to go to 500 tons per day, it would produce enough heat for about 5,100 homes a day and lower the city’s carbon footprint by 90,000 metric tons per year, which is the equivalent of taking about 19,000 vehicles off the road.”

Although the project seems promising and technically feasible, two questions remain: What is the cost of the added load on the wastewater treatment plant, and can a sustainable business model be put in place?

The forthcoming demonstration project is intended to provide some answers. There is ample evidence that adding food to wastewater digesters leads to increased production of higher-quality biogas, Fiore says.

However, the treatment plant may have to contend with other problems related to trace compounds in the biogas, problems that are much less well-understood. These include increased nitrogen loads, production of odiferous compounds and struvite (a precipitate that can clog pipes, pumps, and equipment), buildup of volatile acids that can prevent the digester running smoothly, and undesirable changes in the quality and characteristics of the residual digestate. DEP is partnering with the New York State Energy Research and Development Authority, Manhattan College, and Bucknell University to study these issues.

**Figure:**
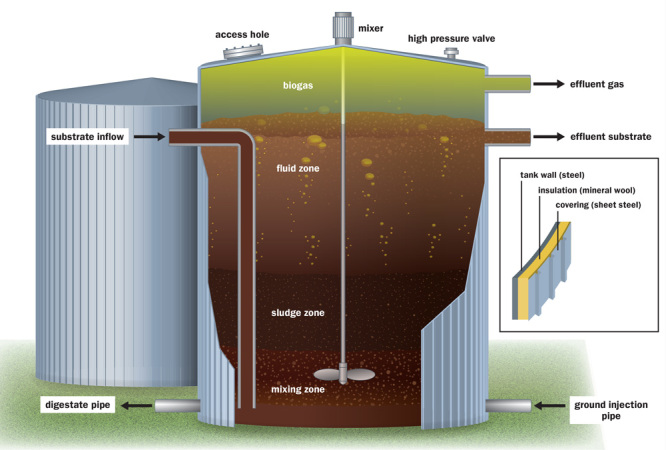
Biodigesters function much the same way as mammalian digestion. Liquefied organic waste (“substrate”) is fed into a sealed tank that’s insulated to maintain a consistent warm temperature. Anaerobic microorganisms degrade the material to produce a mixture of gases and a residual sludge (“digestate”). The composition of the substrate is an important determinant of the quality of the gas and digestate. © Jane Whitney

## New Opportunities

In response to the new Massachusetts ban, John Fischer, branch chief for commercial waste reduction and planning at the state Department of Environmental Protection, says several companies and cities have applied for grants or low-interest loans from the state to build anaerobic digesters. He says there’s room for more digesters in the marketplace. But the flow of food waste is finite, he adds, and once adequate infrastructure is in place to handle that flow, there won’t be room for much more.

Meanwhile, large food retailers are beginning to take advantage of anaerobic digestion. In 2013 Kroger Company became the first grocery company to convert its food waste into energy when it built a digester capable of handling 150 tons of food waste per day.^11^ All groceries that are not sold or donated are shipped to the digester, which is housed at the company’s distribution center in Compton, California. More recently, the Stop & Shop Supermarket Company broke ground on an anaerobic digester facility in Freetown, Massachusetts, with a planned capacity of 95 tons per day and expected opening date of early 2016.^12^

Universities also see the benefit of using food waste for energy. On 22 April 2014 the University of California, Davis, opened an anaerobic digestion facility with a daily capacity of converting 50 tons of food waste from the campus and area restaurants into 12,000 kWh of energy.^13^ The facility features advanced anaerobic digestion technology developed by Ruihong Zhang, a professor of biological and agricultural engineering at the university, and commercialized by Sacramento-based tech company CleanWorld. The patented technology increases the efficiency of the basic anaerobic digestion process. The operators estimate the facility will divert 20,000 tons of food waste from local landfills each year.^13^

In late 2013 a group of researchers from the University of Cincinnati initiated an anaerobic digestion project to produce not just biogas and fertilizer solids but also biodiesel. Tim Keener, a professor in the university’s Department of Biomedical, Chemical, and Environmental Engineering, says that school’s project is unique because it couples anaerobic digestion with algaculture, extracting the carbon dioxide portion of the biogas to grow algae. Lipids isolated from the algae are then converted to biodiesel fuel. The researchers hope to team with the city of Cincinnati, which has expressed interest in a food waste diversion program, to create a facility utilizing the technology.

Thomas Trabold, director of the Center for Sustainable Mobility at the Golisano Institute for Sustainability, Rochester Institute of Technology, is a strong proponent of food waste conversion via anaerobic digestion, but he also points out that the outflow of gas and digestate must be properly managed. For instance, the gas must be captured, not leaked to the atmosphere, and digestate may contain chemical and biological contaminants that must be controlled carefully if the material is to be used as a fertilizer.^14^

Analyses of waste-to-energy opportunities in New York State by Trabold and colleagues indicate significant potential for and interest in expansion.^15^^,^^16^^,^^17^ Trabold predicts a growing role for food waste power generation in the United States. “I definitely think [biodigesters will] become part of the grid, but how significantly remains to be seen,” he says. “It’s going to be a fairly small percentage of the total energy demand, but I think it will become a fairly important part of the portfolio going forward as we move toward more of a distributed power generation model, instead of having a small number of big centralized power plants.”

Fiore adds that anaerobic digestion is also a relatively simple way to affordably implement a steady supply of renewable energy into dense urban areas. The beauty of biogas, he says, is that it helps address energy security by taking advantage of local resources, while fostering the resilience of critical infrastructure and reducing harmful emissions.
